# Supplementary material for: Crosstalk between CST and RPA regulates RAD51 activity during replication stress
Source: Nat Commun. 2021 Nov 5;12:6412. doi: 10.1038/s41467-021-26624-x (PMC8571288; doi:10.1038/s41467-021-26624-x)
Supplement: Supplementary file 1 — Supplementary Information [file 41467_2021_26624_MOESM1_ESM.pdf]

Supplementary Information

Crosstalk between CST and RPA regulates RAD51 activity during replication stress

Kai-Hang Lei[1], Han-Lin Yang[2], Hao-Yen Chang[1], Hsin-Yi Yeh[1], Dinh Duc Nguyen[3], Tzu-Yu Lee[2], Xinxing Lyu[3], Megan Chastain[4], Weihang Chai[3], Hung-Wen Li[2*], Peter Chi[1, 5*]

[1]Institute of Biochemical Sciences, National Taiwan University, Taipei, Taiwan
[2]Department of Chemistry, National Taiwan University, Taipei, Taiwan
[3]Department of Cancer Biology, Cardinal Bernardin Cancer Center, Loyola University Chicago Stritch School of Medicine, Maywood, IL 60153, USA
[4]Office of Research, Washington State University, Spokane, WA 99202, USA
[5]Institute of Biological Chemistry, Academia Sinica, Taipei, Taiwan

These authors contributed equally: Kai-Hang Lei, Han-Lin Yang
[*]e-mail: hwli@ntu.edu.tw; peterhchi@ntu.edu.tw

This PDF file includes:
Supplementary Table 1
Supplementary Figures 1 to 7

Supplementary Table 1. Oligonucleotides used for strand exchange, electron microscopy, DNA pulldown, smFRET, CoSMoS, affinity pulldown, and D-loop formation experiments	
Experiments	DNA orientation 5’→3’
**1. Strand exchange & Electron microscopy**	Asterisk (*) indicates ^{32}P-labeled DNA.
**80 nt ssDNA**	
Oligo 1 (80 nt)	TTATGTTCATTTTTTATATCCTTTACTTTATTTTCTC TGTTTATTCATTTACTTATTTTGTATTATCCTTATC TTATTTA
**40 bp dsDNA**	
Oligo 2 (40 nt)	*TAATACAAAATAAGTAAATGAATAAACAGAGAA AATAAAG
Oligo 3 (40 nt)	CTTTATTTTCTCTGTTTATTCATTTACTTATTTTGTA TTA
**2. DNA pulldown**	
Biotin-Oligo 1 (80 nt)	**Biotin-** TTATGTTCATTTTTTATATCCTTTACTTTATTTTCTCT GTTTATTCATTTACTTATTTTGTATTATCCTTATCTTAT TTA
**3. EMSA**	
Cy3-Oligo 4 (55 nt)	**Cy3-**TTAGAGCTTAATTGCTGAATCTGGTGCTGTGG GTGAACCTGCAGGTGGGCAAAGA
**4. Single molecule FRET**	
**35 nt ssDNA overhang**	
Oligo 5 (60 nt)	ACGCT-**Cy3**-GCCGAATTCTACCAGTGCCT-**Cy5**-TGCT AGGACATCTTTGCCCACCTGCAGGTTCACCC
Oligo 6 (25 nt)	**Biotin**-GGGTGAACCTGCAGGTGGGCAAAGA
**dT(13+47) ssDNA overhang**	
Oligo 7 (60 nt)	TGGCGACGGCAGCGAGGC$(T)_{13}$-Cy3-$(T)_{47}$
Oligo 8 (18 nt)	Cy5-GCCTCGCTGCCGTCGCCA-**Biotin**
**5. CoSMoS Assay**	
**80 nt ssDNA overhang**	
Oligo 9 (98 nt)	TGGCGACGGCAGCGAGGCTTATGTTCATTTTTTAT ATCCTTTACTTTATTTTCTCTGTTTATTCATTTACTT ATTTTGTATTATCCTTATCTTATTTA
Oligo 10 (18 nt)	Cy3-GCCTCGCTGCCGTCGCCA-**Biotin**

**6. Affinity pulldown**	
**30 nt ssDNA**	
Oligo 11 (30 nt)	TTATGTTCATTTTTTATATCCTTTACTTTA
**7. D-loop formation assay**	Asterisk (*) indicates ^{32}P-labeled DNA.
**90 nt ssDNA**	
Oligo 12 (90 nt)	***AAATCAATCTAAAGTATATATGAGTAAACTTGGTC TGACAGTTACCAATGCTTAATCAGTGAGGCACCTAT CTCAGCGATCTGTCTATTT

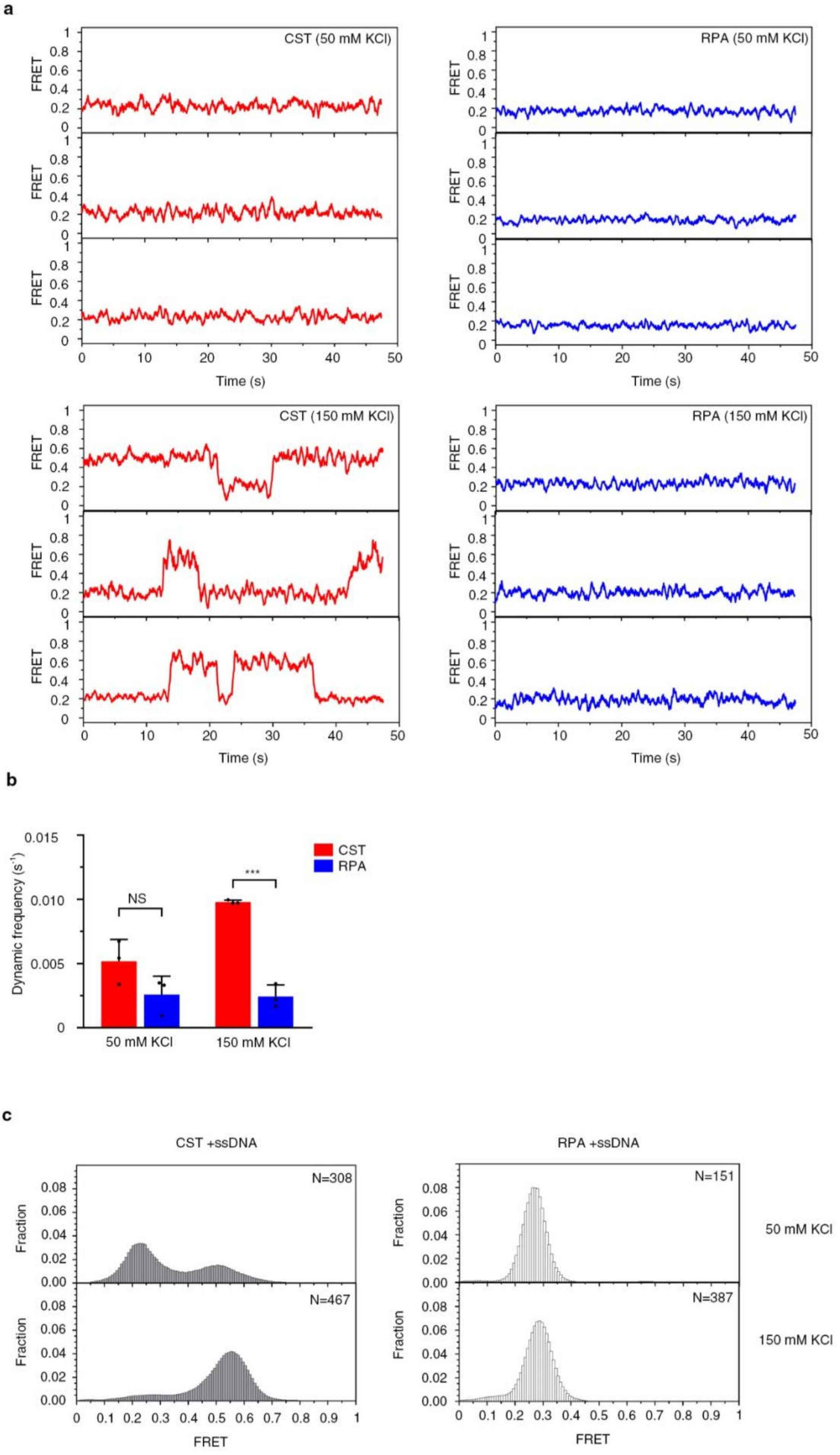
a
CST (50 mM KCl)
RPA (50 mM KCl)
CST (150 mM KCl)
RPA (150 mM KCl)
FRET
Time (s)
b
Dynamic frequency (s⁻¹)
CST
RPA
NS
***
50 mM KCl
150 mM KCl
c
CST +ssDNA
RPA +ssDNA
N=308
N=467
N=151
N=387
Fraction
FRET
50 mM KCl
150 mM KCl

Supplementary Fig. 1. The smFRET experiment showing that CST is dynamic on ssDNA in high ionic strength solution.

a The smFRET time traces of CST or RPA binding to DNA were measured under different KCl conditions by using a 35 nt ssDNA overhang substrate. The CST smFRET time traces are plotted in red (left), whereas those for RPA are in blue (right). In 50 mM KCl (upper panel), both CST- and RPA-bound DNA have smFRET values of ~ 0.2. In 150 mM KCl (lower panels), RPA remains stable and the CST smFRET value dynamically transitions between ~ 0.2 and ~ 0.55, representing protein-bound and DNA-only states, respectively.

b CST presents more transitions between protein-bound and DNA-only states in high ionic strength solution. Dynamic frequency is defined as the number of transitions per second per molecule. Data represent mean ± S.D. calculated from three independent experiments. NS, not significant, ***$P<0.001$, as calculated by unpaired t test (two tailed) assuming that both datasets are Gaussian distributions and have the same S.D.

c Differences in the smFRET histograms reveal the effect of altered salt conditions on the DNA-binding ability of CST and RPA, using the dT (13+47) ssDNA overhang substrate. The smFRET histograms for CST are plotted in gray (left), whereas those for RPA are in white (right). Both CST- and RPA-bound ssDNA display smFRET values of ~ 0.2 in 50 mM KCl (upper panel). In 150 mM KCl (lower panel), RPA-bound ssDNA retains the same smFRET value of ~ 0.2, whereas the smFRET value of CST-bound ssDNA changes to ~0.55. N values represent the number of individual molecules collected from three independent experiments and are displayed in the upper right corner.

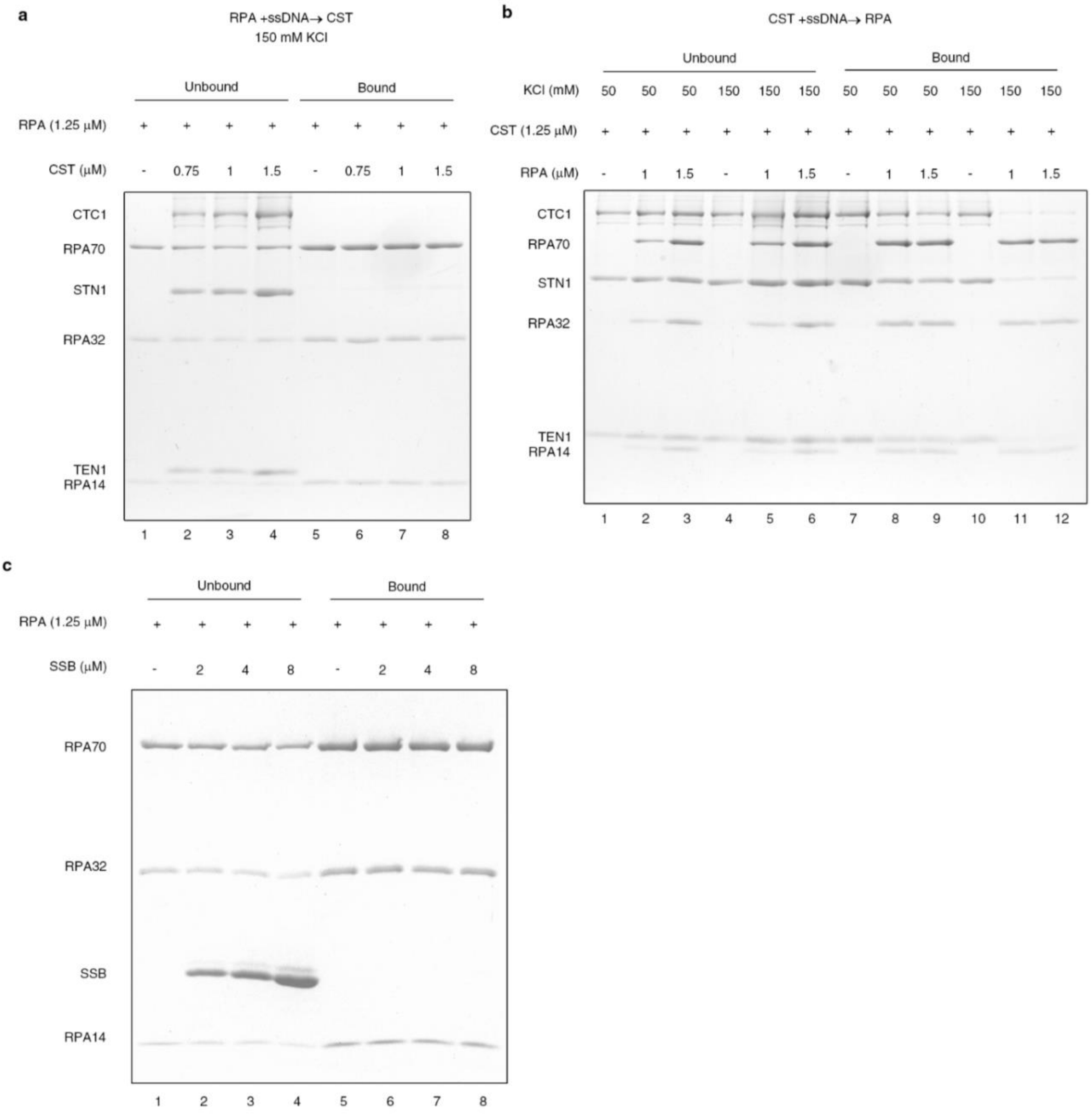

Supplementary Fig. 2. Co-existence of CST with RPA on the same ssDNA under different ionic strengths.

a A ssDNA pulldown assay. RPA was preincubated with magnetic ssDNA beads and then the indicated amounts of CST were added under the condition of 150 mM KCl. The unbound and bound fractions from the reaction were analyzed by 15% SDS-PAGE with Coomassie blue staining. N = 3 biologically independent experiments.

b CST was preincubated with magnetic ssDNA beads and then the indicated amounts of RPA were added under the condition of 50 or 150 mM KCl. N = 3 biologically independent experiments.

c *E. coli* single-stranded binding protein (SSB) cannot bind to RPA-coated ssDNA under the

condition of 50 mM KCl. Excessive RPA was preincubated with a biotinylated 80-nt ssDNA linked to magnetic streptavidin beads. Then *E. coli.* SSB was added and the ssDNA and its associated proteins were captured using a magnetic bead separator. The unbound and bound fractions from the reaction were analyzed by 15% SDS-PAGE with Coomassie blue staining. N = 3 biologically independent experiments.

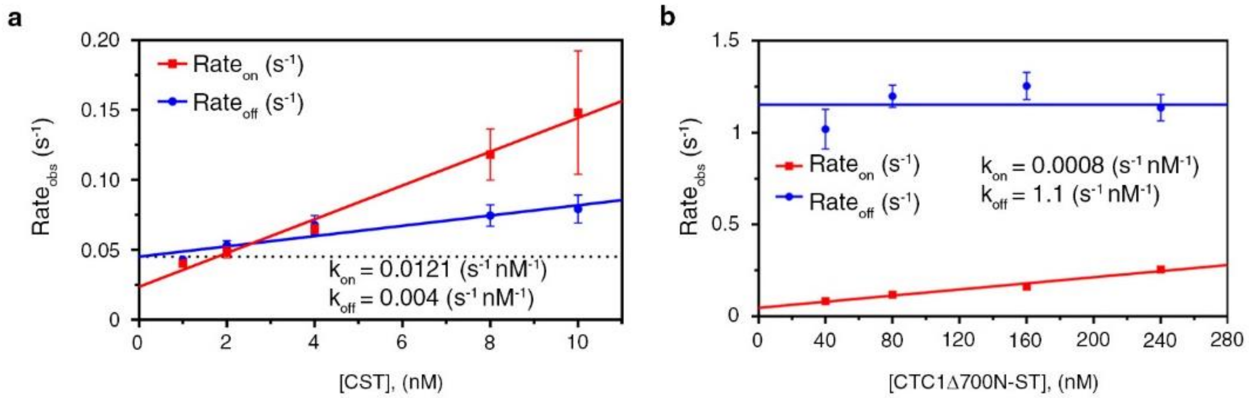

Supplementary Fig. 3. CST exhibits facilitated dissociation.

a Association and dissociation of wild-type CST on the TG-rich 15-nt ssDNA substrate. We determined association rates and dissociation rates by fitting smFRET time traces. Both rates are dependent on CST concentrations, with rate constants of 0.012 s^{-1} nM^{-1} and 0.004 s^{-1} nM^{-1}, respectively. The off-rate dependency of CST concentrations implies that CST exhibits a property of facilitated dissociation. Data represent mean ± S.D. calculated from three independent experiments.

b Similar experiment for CTC1Δ700N-ST. The association rate constant was determined to be 0.0008 s^{-1} nM^{-1}. The off-rate constant for CTC1Δ700N-ST is independent of CST concentration and was determined to be 1.1 s^{-1} nM^{-1}. The CTC1Δ700N-ST dissociation constant was calculated as ~1.4 μM. Data represent mean ± S.D. calculated from three independent experiments.

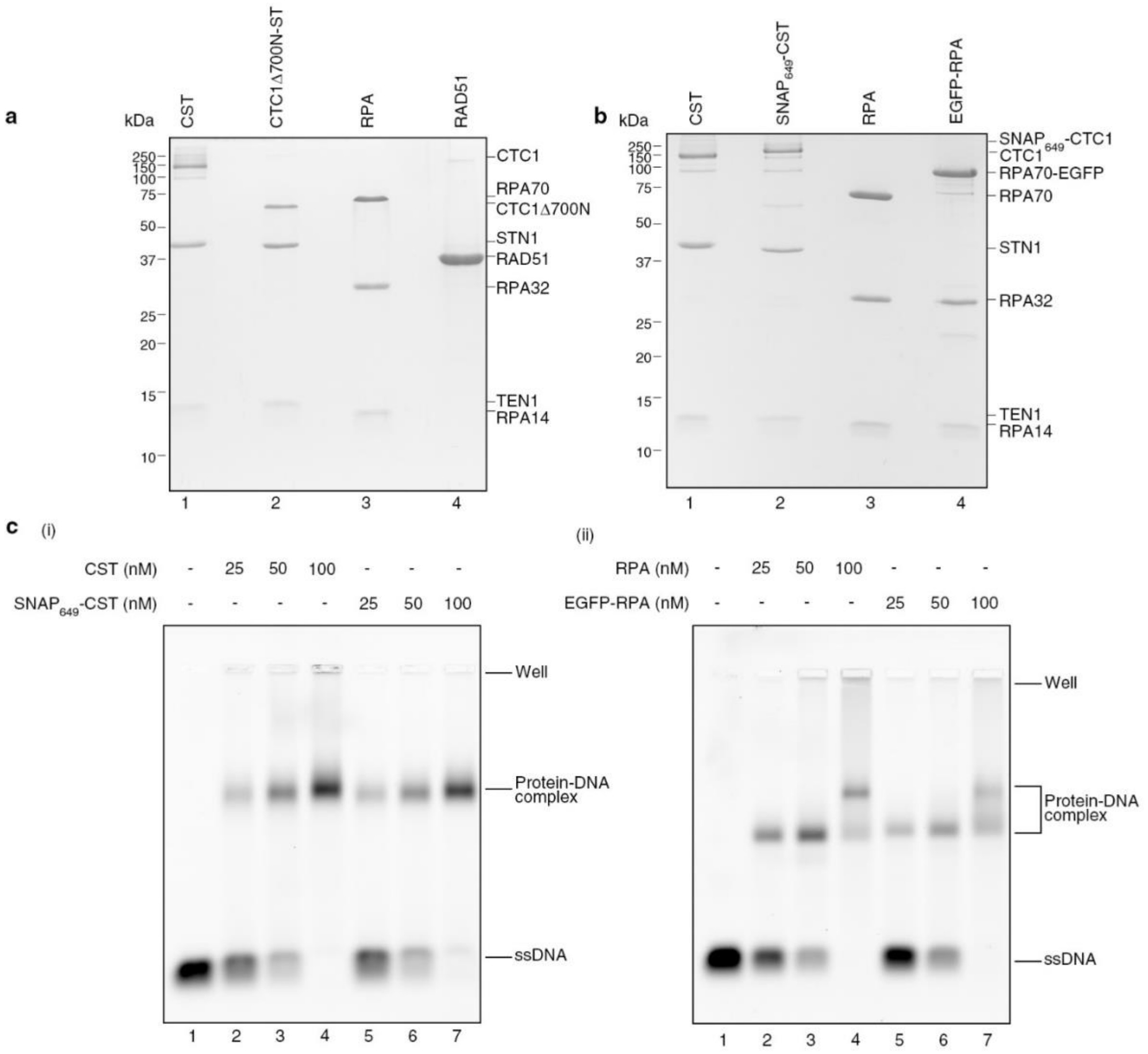

Supplementary Fig. 4. Purified recombinant proteins and their DNA-binding ability.

a Purified human CST, CTC1Δ700N-ST, RPA, and RAD51 proteins were resovled by 15% SDS-PAGE and stained with Coomassie blue. N = 3 biologically independent experiments.

b Purified human $SNAP_{649}$-CST and EGFP-RPA proteins were resolved by 15% SDS-PAGE and stained with Coomassie blue. N = 3 biologically independent experiments.

c The DNA-binding ability of CST and $SNAP_{649}$-CST (i), and RPA and EGFP-RPA (ii) was deteremeined by EMSA. N = 3 biologically independent experiments.

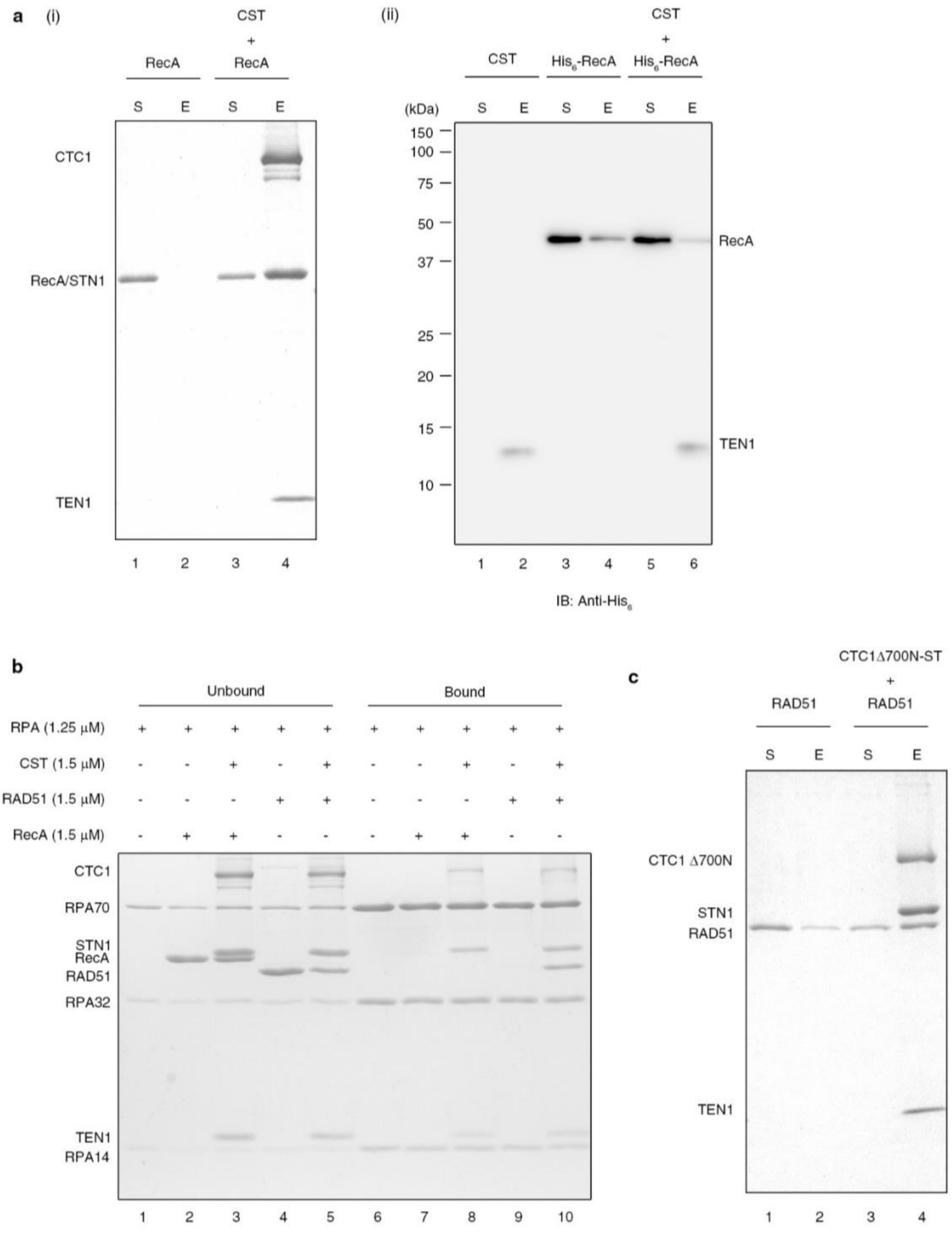

Supplementary Fig. 5. Physical interactions between CST variants and recombinases.

a (i) Affinity pulldown assay. Flag-CTC1-STN1-TEN1-His_6 (1 μM) was incubated with RecA (1 μM), followed by incubation with His-Tag Dynabeads to capture CST and associated proteins using a magnetic bead separator. The supernatant (S) and eluate (E) were analyzed. RecA alone is shown as a control. Note that RecA presents a similar size profile to STN1 in 15% SDS-PAGE. To solve this issue, we performed affinity pulldown (ii) in which Flag-CTC1-STN1-TEN1-His_6 (0.25 μM)

was incubated with His_6-RecA (0.25 μM), followed by incubation with anti-FLAG M2 affinity gel to capture CST and associated proteins. The supernatant (S) and eluate (E) were analyzed by immunoblotting with anti-His antibody. CST and RecA alone are shown as a control. N = 3 biologically independent experiments.

b For ssDNA pulldown analysis, RPA was preincubated with magnetic ssDNA beads. Then, CST and RAD51 or RecA were added to complete the reaction under the condition of 50 mM KCl. The unbound and bound fractions from the reaction were analyzed by 15% SDS-PAGE with Coomassie blue staining. N = 3 biologically independent experiments.

c Flag-CTC1Δ700N-STN1-TEN1-His_6 (1 μM) was incubated with RAD51 (1 μM) for affinity pulldown analysis. His-tag Dynabeads were used to capture the CTC1Δ700N-ST and associated proteins. RAD51 alone is shown as a control. N = 3 biologically independent experiments.

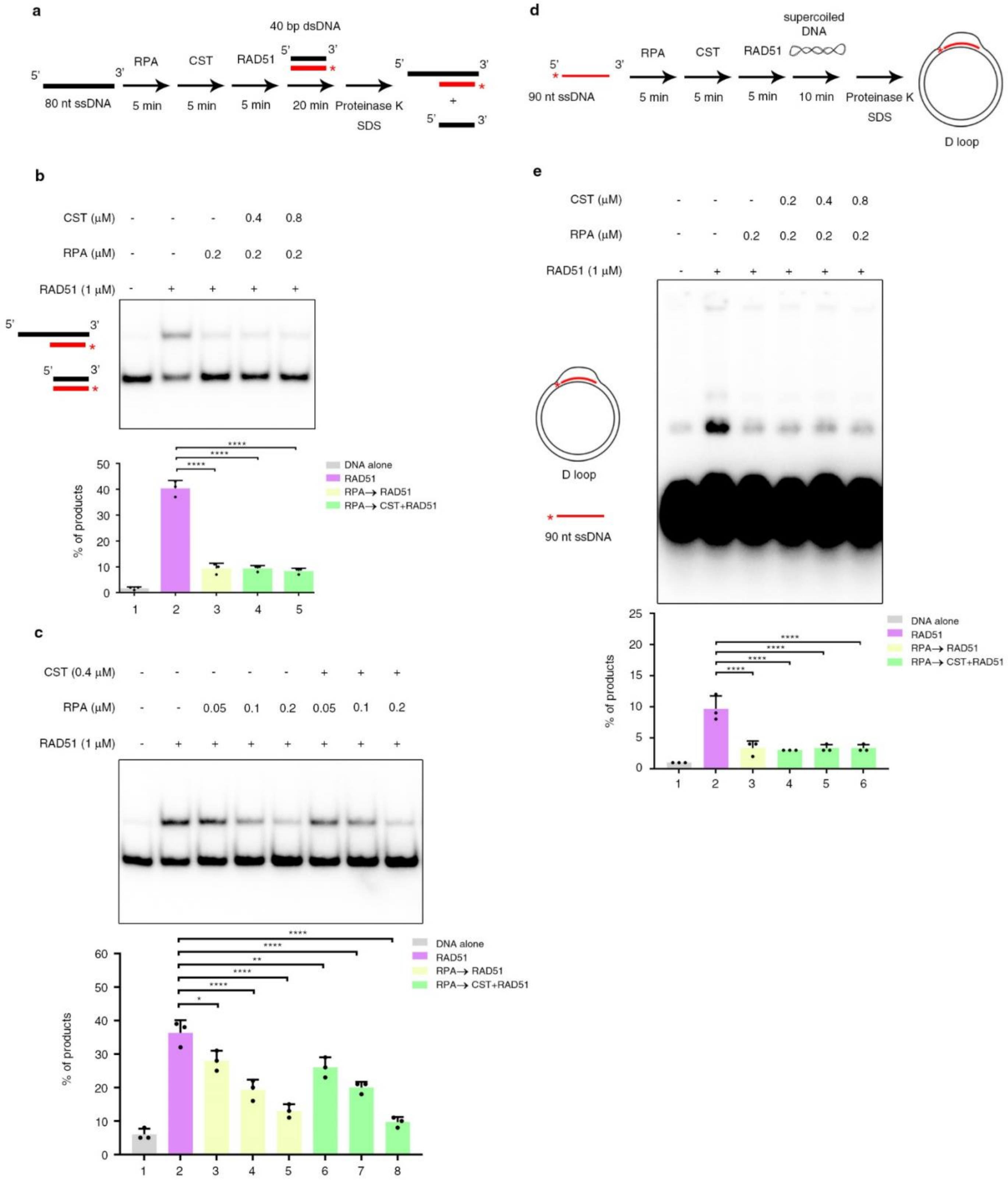

a
40 bp dsDNA
5' 3'
80 nt ssDNA
RPA
5 min
CST
5 min
RAD51
5 min
20 min
Proteinase K
SDS
+
b
CST (μM) - - - 0.4 0.8
RPA (μM) - - 0.2 0.2 0.2
RAD51 (1 μM) - + + + +
% of products
DNA alone
RAD51
RPA→ RAD51
RPA→ CST+RAD51
1 2 3 4 5
c
CST (0.4 μM) - - - - - + + +
RPA (μM) - - 0.05 0.1 0.2 0.05 0.1 0.2
RAD51 (1 μM) - + + + + + + +
% of products
1 2 3 4 5 6 7 8
d
supercoiled DNA
90 nt ssDNA
RPA
5 min
CST
5 min
RAD51
5 min
10 min
Proteinase K
SDS
D loop
e
CST (μM) - - - 0.2 0.4 0.8
RPA (μM) - - 0.2 0.2 0.2 0.2
RAD51 (1 μM) - + + + + +
D loop
90 nt ssDNA
% of products
1 2 3 4 5 6

Supplementary Fig. 6. CST lacks the ability to enhance RAD51 activity using RPA-coated ssDNA.

a Schematic of the DNA strand-exchange assay that we used to examine the mediating activity of CST. The ^{32}P-labeled DNA is marked by an asterisk.

b RPA-bound ssDNA was used as the substrate to monitor RAD51-mediated strand-exchange activity in the presence or absence of the indicated amounts of CST under the condition of 150 mM KCl. Note that 5 mM $CaCl_2$ was used to stimulate RAD51 activity. Quantitative data are shown. Data represent mean ± S.D. calculated from three independent experiments. NS, not significant, ****P<0.0001 as calculated by one-way ANOVA with Tukey's post hoc test.

c The indicated amounts of RPA were used to form RPA-bound ssDNA as substrate to measure RAD51-mediated strand-exchange activity in the presence of CST under the condition of 150 mM KCl. Note that 5 mM $CaCl_2$ was used to stimulate RAD51 activity. Quantitative data are shown. Data represent mean ± S.D. calculated from three independent experiments. NS, not significant; *P<0.05; **<0.01; ****P<0.0001 as calculated by one-way ANOVA with Tukey's post hoc test.

d Schematic of the D-loop formation assay. The ^{32}P-labeled 90-nt ssDNA is marked by an asterisk.

e RPA-bound ssDNA was used as the substrate to monitor RAD51-mediated DNA pairing and strand-exchange activity in the presence or absence of the indicated amounts of CST under the condition of 150 mM KCl. Note that 10 mM $CaCl_2$ was used to stimulate RAD51 activity. Quantitative data are shown. Data represent mean ± S.D. calculated from three independent experiments. NS, not significant; ****P<0.0001 as calculated by one-way ANOVA with Tukey's post hoc test.

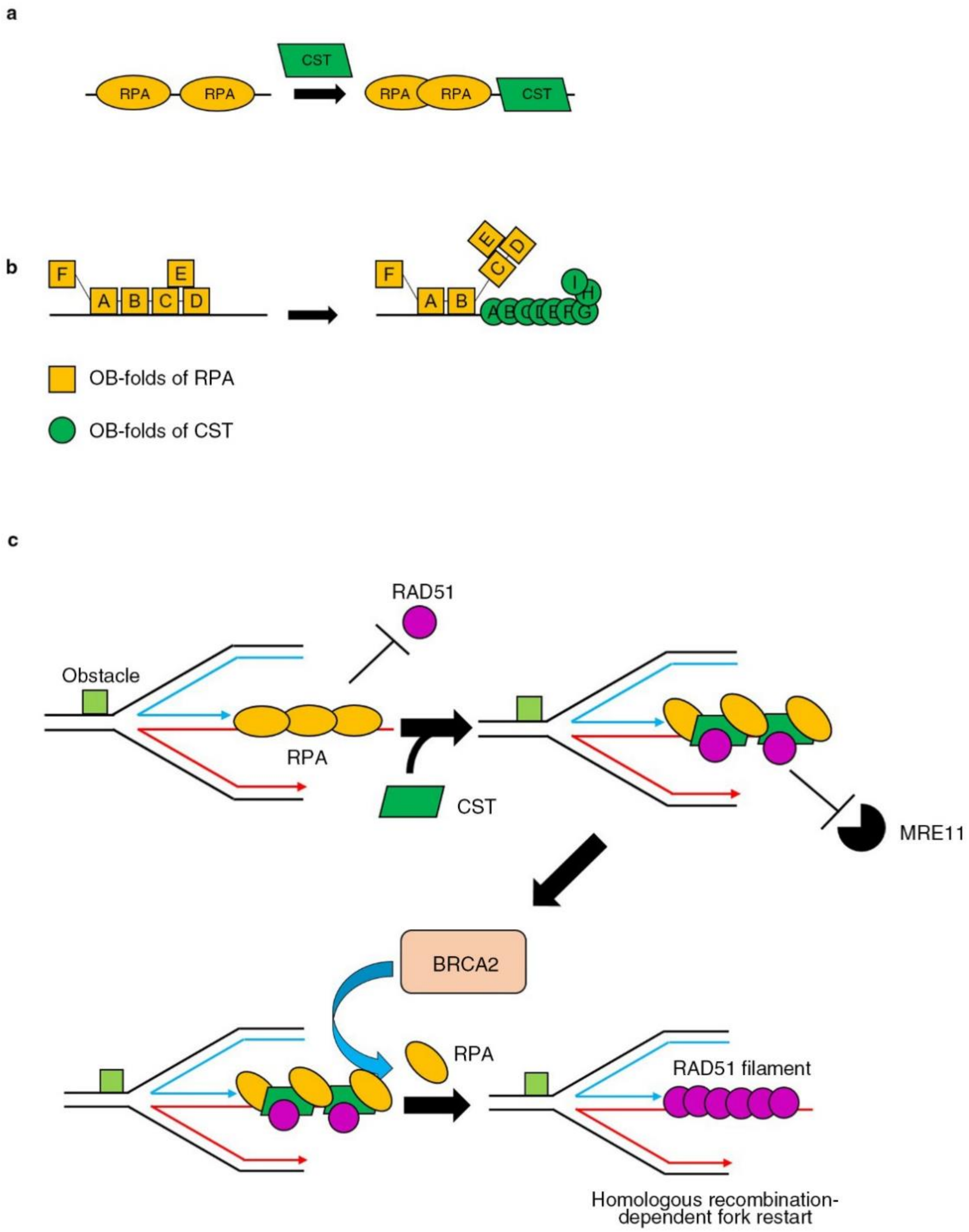

Supplementary Fig. 7. Proposed models for the crosstalk between RPA, CST, and RAD51 in response to replication stress.

a, b Proposed models for how CST coexists with RPA on ssDNA.

c Under replication stress, the exposed ssDNA intermediates generated from remodeling of stalled/collapsed forks act as substrates for the OB-fold-containing RPA and CST complexes. RPA acts as "first responder" to bind ssDNA due to its high ssDNA-binding affinity and high abundance

in cells. RPA protects the ssDNA intermediates from nuclease attack and triggers DNA damage responses[3,4]. However, RPA-coated ssDNA prevents RAD51 from loading, which may influence the formation and protection of reversed forks and restart of stalled/collapsed forks. Accordingly, RAD51 access to RPA-coated ssDNA represents an important regulatory node. Our study shows that CST physically interacts with RAD51 and, most importantly, that it can target and tether RAD51 to RPA-coated ssDNA via a facilitated dissociation mechanism. Notably, CST does not act as mediator for RAD51-mediated strand exchange. Other mediators, such as BRCA2, likely act as prerequisites for RPA displacement and RAD51 filament assembly.
