# Supplementary material for: Crosstalk between CST and RPA regulates RAD51 activity during replication stress
Source: Nat Commun. 2021 Nov 5;12:6412. doi: 10.1038/s41467-021-26624-x (PMC8571288; doi:10.1038/s41467-021-26624-x)
Supplement: Supplementary file 3 — Reporting Summary [file 41467_2021_26624_MOESM3_ESM.pdf]

Corresponding author(s): Peter Chi, Hung-Wen Li

Last updated by author(s): 2021/9/22

# Reporting Summary

Nature Research wishes to improve the reproducibility of the work that we publish. This form provides structure for consistency and transparency in reporting. For further information on Nature Research policies, see our Editorial Policies and the Editorial Policy Checklist.

## Statistics

For all statistical analyses, confirm that the following items are present in the figure legend, table legend, main text, or Methods section.

| n/a | Confirmed | |
|---|---|---|
| ☐ | ☒ | The exact sample size (*n*) for each experimental group/condition, given as a discrete number and unit of measurement |
| ☐ | ☒ | A statement on whether measurements were taken from distinct samples or whether the same sample was measured repeatedly |
| ☐ | ☒ | The statistical test(s) used AND whether they are one- or two-sided<br>*Only common tests should be described solely by name; describe more complex techniques in the Methods section.* |
| ☒ | ☐ | A description of all covariates tested |
| ☐ | ☒ | A description of any assumptions or corrections, such as tests of normality and adjustment for multiple comparisons |
| ☐ | ☒ | A full description of the statistical parameters including central tendency (e.g. means) or other basic estimates (e.g. regression coefficient) AND variation (e.g. standard deviation) or associated estimates of uncertainty (e.g. confidence intervals) |
| ☐ | ☒ | For null hypothesis testing, the test statistic (e.g. *F*, *t*, *r*) with confidence intervals, effect sizes, degrees of freedom and *P* value noted<br>*Give P values as exact values whenever suitable.* |
| ☒ | ☐ | For Bayesian analysis, information on the choice of priors and Markov chain Monte Carlo settings |
| ☒ | ☐ | For hierarchical and complex designs, identification of the appropriate level for tests and full reporting of outcomes |
| ☒ | ☐ | Estimates of effect sizes (e.g. Cohen's *d*, Pearson's *r*), indicating how they were calculated |

*Our web collection on statistics for biologists contains articles on many of the points above.*

## Software and code

Policy information about availability of computer code

| | |
|---|---|
| Data collection | Software for a particular experiment was used as follows:<br>Image Lab software 6.0 (Bio-Rad): DNA pulldown assay and affinity pulldown assay.<br>Quantity One software 4.6.9 (Bio-Rad): DNA strand exchange and D-loop formation assay.<br>VisionWorks LS Software 8.6 (UVP) & ImageQuant LAS4000 (GE): immunoblotting images.<br>Amersham Typhoon 2.0 : EMSA.<br>Gatan model 782 Erlangshen ES500W: Electron microscopy image.<br>LabVIEW (2016): smFRET & CoSMoS assays. smFRET image data were collected by a home-built LabView program which was available from the corresponding author upon reasonable request.<br>LabVIEW (2016): CoSMoS images were gathered under the open-source LabView program which was available at Jeff Gelles's lab GitHub (https://github.com/gelles-brandeis/Glimpse).<br>ZEN (Zeiss): Immunostaining & PLA image.<br>- For Immunostaining: AxioVision<br>- For PLA image: ZEN 3.0 (blue edition) |
| Data analysis | Quantitative analyses for phosphorimages were performed using Quantity One software 4.6.9 (Bio-Rad).<br>Quantitative analyses for DNA pulldown, affinity pulldown, and RAD51 filament measurement were performed using Image J 1.52a.<br>smFRET intensity data were extracted by IDL software 8.3 (ITT Vis) and FRET efficiency data were performed using MATLAB (r2016a). All codes are available from the corresponding author upon reasonable request.<br>CoSMoS analysis was performed by open-source MATLAB program, imscroll, which was available at Jeff Gelles's lab GitHub (https://github.com/gelles-brandeis/CoSMoS_Analysis).<br>Immunostaining & PLA image analyses were performed using ZEN (Zeiss).<br>- For Immunostaining: AxioVision<br>- For PLA image: ZEN 3.0 (blue edition) |

All statistical tests were performed using GraphPad Prism 7&9 ( GraphPad Software).

For manuscripts utilizing custom algorithms or software that are central to the research but not yet described in published literature, software must be made available to editors and reviewers. We strongly encourage code deposition in a community repository (e.g. GitHub). See the Nature Research guidelines for submitting code & software for further information.

## Data

Policy information about availability of data

All manuscripts must include a data availability statement. This statement should provide the following information, where applicable:
- Accession codes, unique identifiers, or web links for publicly available datasets
- A list of figures that have associated raw data
- A description of any restrictions on data availability

All relevant data are described in the Supplementary Information. The figure source data of the figure are provided as a Source Data file. Any additional data related to this paper are available from the corresponding author upon request.

# Field-specific reporting

Please select the one below that is the best fit for your research. If you are not sure, read the appropriate sections before making your selection.

☒ Life sciences ☐ Behavioural & social sciences ☐ Ecological, evolutionary & environmental sciences

For a reference copy of the document with all sections, see nature.com/documents/nr-reporting-summary-flat.pdf

# Life sciences study design

All studies must disclose on these points even when the disclosure is negative.

| | |
|---|---|
| Sample size | No statistical methods were used to predetermine sample size. For PLA assay, The number of cells (n=191-297) is sufficient for analysis based on the established practices in the field (Wooten et al.2019, Nat Struct Mol Biol 26, 732–743; Teng et al. 2021, Nat Commun 12, 3887).<br>For single molecule assay, the sample size was determined by the number of fluorescent DNA molecule within the microscope field of view.<br>For EM analysis, the RAD51 filament number (n=348-398) is enough to analyze the length based on the studies in the field. (Dupaigne et al. 2008, PLOS ONE 3(11): e3643; Liu et al. 2011, Nature 479, 245–248; Špírek, et al. 2018, Nucleic Acids Res, 46(8):3967-3980)<br>For biochemical assay, three repeats are sufficient for evaluating the spread data in this field (Zhao et al. 2017, Nature 550, 360–365) |
| Data exclusions | No data were excluded from the analysis. |
| Replication | All experimental findings were reliably reproduced. The data were analyzed from three independent experiments. |
| Randomization | The fluorescence microscope was set to randomly capture images. And the electron microscopy images were captured randomly. No randomization was used for biochemical assay. |
| Blinding | For EM analysis, samples were blind to analyze to avoid any bias in the measurement and statistical analysis. For the other experiments, since the differences between samples were obvious, blinding was not applicable. |

# Reporting for specific materials, systems and methods

We require information from authors about some types of materials, experimental systems and methods used in many studies. Here, indicate whether each material, system or method listed is relevant to your study. If you are not sure if a list item applies to your research, read the appropriate section before selecting a response.

## Materials & experimental systems

| n/a | Involved in the study |
|---|---|
| ☐ | ☒ Antibodies |
| ☐ | ☒ Eukaryotic cell lines |
| ☒ | ☐ Palaeontology and archaeology |
| ☒ | ☐ Animals and other organisms |
| ☒ | ☐ Human research participants |
| ☒ | ☐ Clinical data |
| ☒ | ☐ Dual use research of concern |

## Methods

| n/a | Involved in the study |
|---|---|
| ☒ | ☐ ChIP-seq |
| ☒ | ☐ Flow cytometry |
| ☒ | ☐ MRI-based neuroimaging |

## Antibodies

| | |
|---|---|
| Antibodies used | Antibodies used for Co-immunoprecipitation:<br>Anti-C-myc antibody (9E10) (Santa cruz, SC-40); 3µg per sample |

Antibodies used for immunoblotting:
Anti-Flag antibody (Sigma,F7425); 1:2000
Anti-c-Myc Antibody (A-14) (Santa Cruz, sc-789); 1:500
Anti-HA tag antibody (Abcam, ab13834); 1:2000
Anti-RPA70 antibody (Bethyl, A300-241A); 1:5000
Anti-polyHistidine antibody (Sigma, H1029) 1:1000

Antibodies used for immunostaining:
Anti-BrdU (Abcam, ab6326); 1:5000
Anti-Flag M2 (Sigma-Aldrich, F1804); 1:500
And anti-RPA pS33 (Bethyl, A300-246A); 1:5000
Alexa 488 anti-rat IgG (ThermoFisher, A11006); 1:500
DyLight 550 anti-mouse IgG (ThermoFisher, 84540); 1:1000
DyLight 649-anti-rabbit IgG(ThermoFisher, 35565); 1:1000

Antibodies used for PLA:
Anti-STN1 (Abcam, ab251856); 1:100
Anti-CTC1,(Abcam, ab230538); 1:100
Anti-RPA32 (Abcam, ab2175); 1:200
PLA Probe Anti-Mouse PLUS (Sigma, DUO82001); 1:5
PLA Probe Anti-Rabbit MINUS (Sigma, DUO82005); 1:5

Validation

Validations of all antibodies are based on the datasheets from the manufactures. The primary antibodies were also validated by overexpression or siRNA knockdown of the target gene.

## Eukaryotic cell lines

Policy information about cell lines

Cell line source(s)

HeLa: CCL-2, from ATCC
293T: CRL-3216, from ATCC
Expi293F: A14527, from ThermoFisher

Authentication

Short tandem repeat profiling

Mycoplasma contamination

Cells were tested negative for mycoplasma contamination.

Commonly misidentified lines
(See ICLAC register)

No commonly misidentified cell lines were used.
